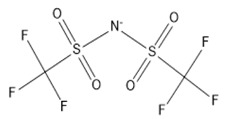# COSMO-RS Based Prediction for Alpha-Linolenic Acid (ALA) Extraction from Microalgae Biomass Using Room Temperature Ionic Liquids (RTILs)

**DOI:** 10.3390/md18020108

**Published:** 2020-02-12

**Authors:** Shiva Rezaei Motlagh, Razif Harun, Dayang Radiah Awang Biak, Siti Aslina Hussain, Rozita Omar, Amal A. Elgharbawy

**Affiliations:** 1 Department of Chemical and Environmental Engineering, Faculty of Engineering, University Putra Malaysia, 43400 UPM, Serdang, Selangor, Malaysia; shiva.rezaei.m@gmail.com (S.R.M.); dradiah@upm.edu.my (D.R.A.B.); aslina@upm.edu.my (S.A.H.); rozitaom@upm.edu.my (R.O.); 2 International Institute for Halal Research and Training (INHART), International Islamic University Malaysia, Gombak, 50728 Kuala Lumpur, Malaysia; amal.elgharbawy@gmail.com

**Keywords:** ionic liquids, COSMO-RS, ALA extraction, solid-liquid extraction, tetramethyl ammonium, Omega-3 PUFAs, capacity at infinite dilution

## Abstract

One of the essential fatty acids with therapeutic impacts on human health is known to be omega-3 polyunsaturated fatty acids (PUFA). More lately, ionic liquids (ILs) have received significant attention among scientists in overcoming the disadvantages of traditional solvents in biomass lipid extraction. However, the large pool of cations and anions possibly accessible will lead to a growing number of innovatively synthesized ILs. Nevertheless, the exhaustive measurement of all these systems is economically impractical. The conductive screening model for real solvents (COSMO-RS) is considered a precious approach with the availability of a few models to predict the characteristics of ILs. This work introduces the estimate of capacity values at infinite dilution for a range of ILs using COSMO-RS software as part of solid-liquid extraction. This favorable outcome presented that the capacity values of the IL molecules are extremely dependent on both anions and cations. Among the 352 combinations of cation/anion tested, short alkyl chain cations coupled with inorganic anions were found to be most efficient and therefore superior in the extraction method. Sulphate-, chloride-, and bromide-based ILs were found to have higher extraction capacities in contrast with the remainders, while propanoate revealed an extraordinary capacity when combined with ethyl-based cations. Eventually, the predicted results from COSMO-RS were validated through the experimentally calculated extraction yield of alpha-linolenic acid (ALA) compound from *Nannochloropsis* sp. microalgae. Three selected ILs namely [EMIM][Cl], [TMAm][Cl], and [EMPyrro][Br] were selected from COSMO-RS for empirical extraction purpose and the validation results pinpointed the good prediction capability of COSMO-RS.

## 1. Introduction

**Figure 1:**
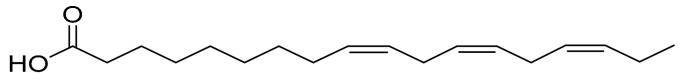
Chemical structure of alpha-linolenic acid (ALA).

Lifestyle-related diseases (e.g., obesity, hyperlipidemia, arteriosclerosis, diabetes mellitus, and hypertension) are complex, where the precise underlying mechanisms are yet to be fully understood [1]. There is growing proof, however, that particular omega-3 PUFAs have positive impacts on human health and may contribute to the prevention of many such chronic diseases in humans [2]. Omega-3 PUFAs are categorized as alpha-linolenic acid (ALA), eicosapentaenoic acid (EPA), and docosahexaenoic acid (DHA). ALA has been reported as the most affordable and sustainable source of PUFA in vegetable oils such as canola oil, soybean oil, flaxseed oil, pumpkin seed oil, perilla seed oil, tofu, and walnut oil [3] and the chemical structure is show in Figure 1. It has revealed positive neuroprotective, anti-inflammatory, and antidepressant effects, and useful for renal issues [4]. 

**Figure 2:**
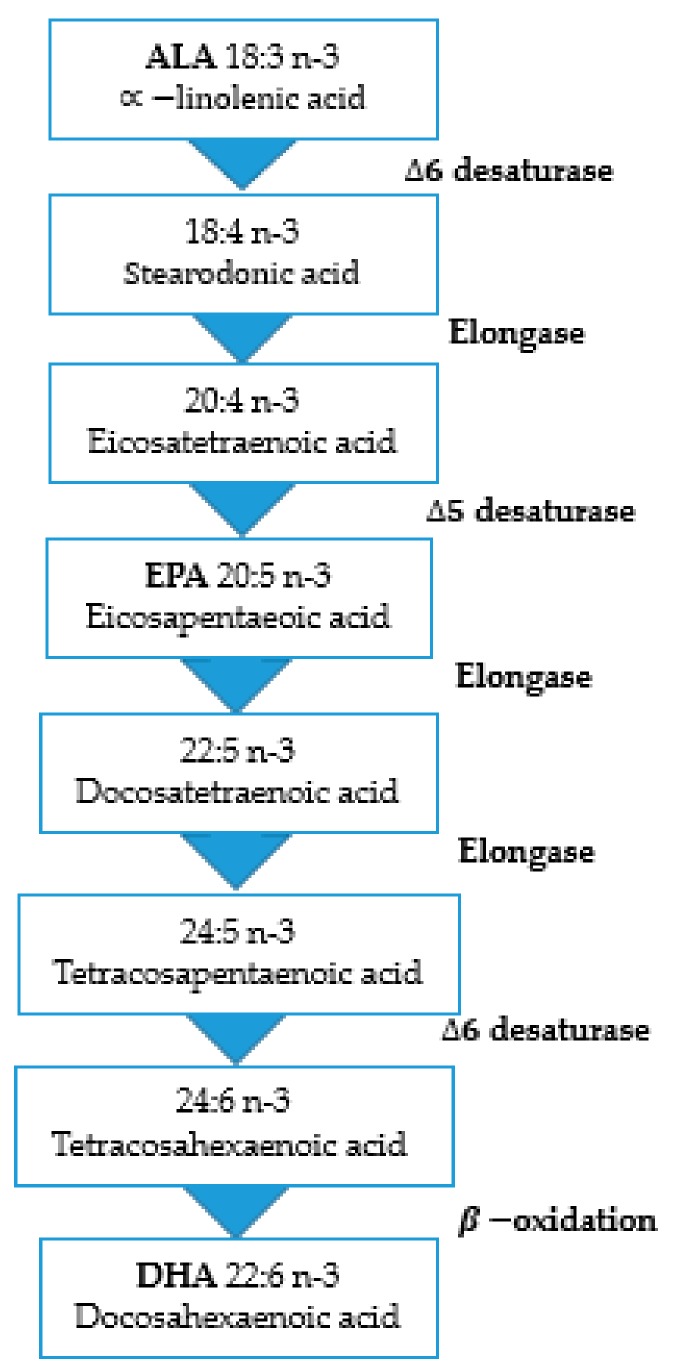
Conventional Δ6-pathway for the biosynthesis of eicosapentaenoic acid (EPA) and docosahexaenoic acid (DHA) polyunsaturated fatty acids by ALA.

From a different perspective, long-chain omega-3 fatty acids such as EPA and DHA are equally crucial nutrients for improving quality of life and decreasing the risk of premature death [4]. They are essential for fetal development, cardiovascular function, and Alzheimer’s disease [5]. Unlike saturated and monounsaturated fatty acids, PUFA cannot be synthesized de novo by mammals and humans, because they lack the required enzymes. Hence, EPA and DHA must be provided through particular foods. These consist primarily of fish, nutraceuticals, and functional foods [6] or ALA body synthesis [5]. The n-3’s fatty acid family has a double-bond beginning from the third carbon’s methyl side. Since mammals do not insert double boundaries closer to the methyl end than the ninth carbon atom (D-9 desaturase), it is not possible to synthesize de novo n-3 fatty acids. As a result, ALA and other fatty acids can synthesize EPA and DHA via chain elongation and desaturation via the omega-3 pathway (Figure 2) [7].

On the other side, ALA is EPA and DHA’s “parent” fatty acid. The human body transforms ALA easily into EPA, but slower into DHA [8]. Nevertheless, bioconversion of ALA to EPA and DHA is limited; therefore, we require adequate dietary intake of long chain omega-3 from external sources like plants, fish, and microalgae. Generally, fish is considered as the main source of PUFAs [9]. However, the quality of fish oil is changing and depends on the type of fish, seasonal time, and place of fishing [10]. Additionally, the application of fish oil in food, infant formulas, or pharmaceuticals have some disadvantages because of their contamination by environmental pollution such as heavy metal accumulation [11]. Therefore, microalgae turned into one of the most important producers of omega-3 PUFA [12]. Generally, the conversion of microalgae into omega-3 PUFAs consists of four steps including microalgae cultivation, harvesting, cell disruption, and lipid extraction [13]. Among these steps, the lipid extraction stage is the most important step to enhance the quality and quantity of microalgae lipid production. Omega-3 PUFA is then generated by a chemical reaction known as transesterification in which fatty acids respond in the presence of appropriate catalysts with methanol [14].

Various extraction methods have been proposed for this purpose, both traditional and nontraditional extraction techniques [15]. Traditional techniques used in the extraction of lipids from biomass have several disadvantages; longer processing time, lower product selectivity, lower extraction efficiency, and laborious. These traditional methods use a large quantity of toxic solvents [16]. However, various alternative techniques such as enzymatic hydrolysis, fractionation, pyrolysis, osmotic shock, and ionic liquid are usually used to obtain microalgal lipids [17].

ILs are organic salts with significantly reduced melting points when compared to traditional inorganic ionic compounds, for example, NaCl [18]. ILs are increasingly being applied in liquid-liquid and solid-liquid extractions [19]. This is because their hydrophobic or hydrophilic characteristics can be customized by cation and/or anion changes [20]. In addition, the implementation of ILs in separations opens up some fresh viewpoints in improving the appropriate process parameters (e.g., selectivity, capacity, relative volatility) by correctly selecting the mixture of cation-anion [21]. Consequently, many researchers have suggested using ILs as a green solvent to extract lipids from biomass in recent years [21,22,23,24,25].

A relatively recent study [23] investigated lipid extraction from microalgae *Chlorella vulgaris* using [BMIM][MeSO_4_] and compared this with traditional methods. They found that the total extractable lipid from *C. vulgaris* by the Soxhlet method and the Bligh and Dyer’s method were 21 and 29 mg g^−1^ dry cell weight (DCW), respectively, whereas 47 mg g^−1^ DCW was achieved with [BMIM][MeSO_4_]. Other researchers [24] extracted fat from *C. vulgaris* using a mixture of methanol and ILs. Lower lipid content of 11.1% was obtained when using the Bligh and Dyer method as compared to [BMIM][CF_3_SO_3_] which was 19.0%. They discovered that ILs’ dipolarity/polarizability and hydrogen bond acidity were more critical than their basic hydrogen bond basicity to efficiently extract lipids from microalgae biomass.

In another study [25], the effect of single ILs was compared with those by organic solvents and IL blends for the extraction of lipids from *C. vulgaris* microalgae. The yield of total lipid using the MeOH:CHCl_3_ (1:2) solvent was 380 mg g^−1^ cell. Among the 12 ILs tested, [EMIM]Ace, [EMIM]DEP, [EMIM]BF_4_, and [EMIM]Cl demonstrated high (>200.0 mg g^−1^ cell) lipid extraction yields. Though the extraction efficiency of [EMIM]EtOSO_3_ and [BMIM]SCN were only 60.5 and 42.7 mg g^−1^ cell, respectively, the output for their blend (a weight ratio of 1:1) was enhanced to 158.2 mg g^−1^ cell. Similarly, while [EMIM]HSO_4_’s lipid extraction yield was only 35.2 mg g^−1^ cell, it was increased up to 200.6 mg g^−1^ cell for its blend with [EMIM]SCN (1:1 weight ratio). By and large, IL mixtures’ synergistic impacts with various anions could enhance the production of lipid extraction from C. *vulgaris* [25].

ILs can be tailored to achieve different physiochemical characteristics by modifying their mixture of cation and anions [26]. The task of experimental solvent screening is often expensive and time-consuming due to the large number of possible solvents that could meet specific requirements [27]. The literature has indicated several options for solvent screening. These generally include heuristic techniques and computer-aided molecular designed (CAMD) to promote solvent selection [28]. Although CAMD can escalate the solvent selection process, group contribution methods such as UNIFAC [29] are still required to evaluate activity coefficients (Ac) and thermodynamic properties in liquid mixtures. However, the required UNIFAC interaction parameters are not always easily accessible. This is particularly difficult when it comes to comparison with the stranger compounds [30] due to the limitation of the data bank. 

The COSMO (COnductor like Screening MOdel) [31] together with Real Solvents (i.e., COSMO-RS) could be used to compute the chemical potential and Ac of any component in a mixture. COSMO-RS is a convenient tool requiring only the molecular structure as input while being still independent of any experimental data [32,33]. Hence, the COSMO-RS does not require experimental data or adjustable coefficients/parameters to estimate the Ac required for phase equilibrium calculations. The calculations can be accomplished solely on the basis of the 3D molecular conformation, obtained from Ab-initio electronic calculations. Therefore, the chemical structure (i.e., the composition and molecular topology) is the only input data set needed in COSMO-RS. In fact, this is the main advantage of COSMO-RS over other thermodynamic approaches such as molecular-based equations of state or Gibbs free energy local composition models which usually employ a number of parameters adjusted to pure-fluid data and/or at least one binary interaction parameter fitted to the mixture data. Besides, COSMO-RS uses a statistical thermodynamics strategy to understand the dissolving phenomena based on the results of quantum chemical calculations. As a result, these capabilities make COSMO-RS a practically superior alternative for the calculation of activity coefficients in ILs. COSMO-RS has been used to generate molecular surface charge distributions and also to calculate activity coefficient at infinite dilution ($Ac^{\infty }$) of solute in the ILs phase. $Ac^{\infty }$ is an important parameter enabling the study of the deviation from ideal behavior in a mixture. Essentially, it discloses the data in a combination related to the nonideality of a chosen species in a mixture. The value defines the extreme scenario i.e., when only solvent–solute interaction contributes to the nonideality. This has practical consequences for both chemical and industrial procedures. The capacity values of ILs, relating to the amount of IL necessary for a successful extraction process, can be obtained with the help of $Ac^{\infty }$ [34]. Both selectivity and capacity are strongly intertwined with solvent–solute interaction effects [33]. The extraction capacity also relies on the volume of the anion and the energy of contact between the cation and the anion [34].

Many researchers have used $Ac^{\infty }$ as the thermodynamic property of solvents to predict the relationships between the solution and the solvents [35]. A stronger solute–solvent interaction is identified by a lower Ac and vice versa [36].

In our previous study [37], COSMO-RS was used to implement the capacity values at infinite dilution in predicting the ability of potential ILs for the extraction of EPA (omega-3) from biomass. We found that [TMAm][SO_4_] showed the highest capacity for EPA extraction among the 352 screened cation/anion combinations studied in our work. With the implementation of this mixture as the solvent in the extraction method, a greater EPA yield is anticipated to be observed. ILs with small anions showed greater capacity and greater charge density relative to their bigger counterparts. They therefore preferred ILs to be used in the applications for extraction. On the other side, when using imidazolium based ILs as validated with experimental information, shorter alkyl chain cations are usually preferred. 

As far as the authors understand, no report has been published on the use of COSMO-RS as a prediction tool to screen the appropriate ILs for the extraction of ALA molecules. Therefore, the Innovation of this study is the use of COSMO-RS for this purpose as a part of solid-liquid extraction. Henceforth, this study is significant not only for a researcher who works on ALA extraction but also it is a new horizon for researchers seeking the extraction of short chain and other long chain fatty acids using ILs. 

Therefore, in this research the goal is to assess COSMO-RS capabilities as the screening method to predict ILs capacity values at infinite dilution of five different types of cation-based ILs with 22 anions as a part of microalgae biomass solid-liquid extraction of ALA. Additionally, the most important and innovative prospect in this study is that it predicts the capacity values of a variety of ILs as the part of solid-liquid extraction from biomass while most related previous studies have been focused on the liquid-liquid extraction process. 

Then, this study aims to validate the predicted results from COSMO-RS by the experimental part. For this purpose, we experimentally calculate the extraction yield of the ALA compound from *Nannochloropsis* sp. microalgae using the selected ILs from COSMO-RS software to compare with ALA capacity values predicted. 

## 2. Results and Discussion

**Figure 3:**
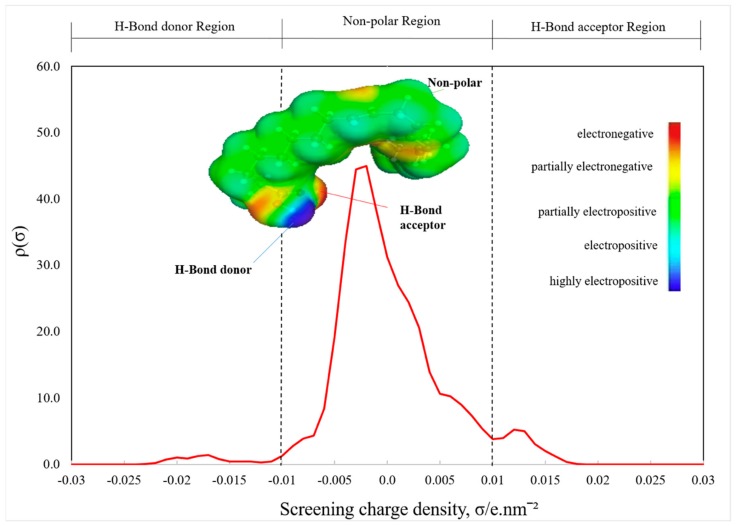
Sigma profiles and sigma surface of the ALA molecule predicted by conductor like screening model for real solvents (COSMO-RS) analysis.

**Figure 4:**
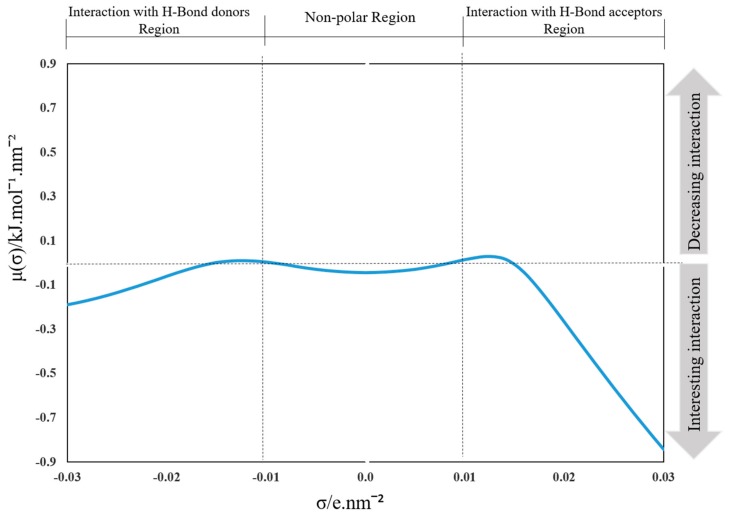
The sigma potential of the ALA compound estimated by COSMO-RS.

The sigma surface coupled with the sigma profile and the sigma potential are shown in Figure 3 and Figure 4. The key terms related to the qualitative description of the ALA molecules (i.e., hydrogen bonding, polarity, and lipophilicity/hydrophilicity) can be simply envisioned with the usage of COSMO-RS 3D screening charge distribution (i.e., sigma surface). 

The *σ*-profile demonstrates the distribution of the electronic polar charge related to a molecular surface. It is portrayed as a possibility plot ρ(s) or histogram, indicating how much of the molecular surface has a certain interval of polarity (*σ*) [31]. The system’s sigma profiles (i.e., ALA molecules) in our study are shown in Figure 3. The COSMO-RS model was utilized to create the sigma profile of the ALA molecules to gain more insight into the impact of cations and anions. 

The ALA *σ*-profile discloses a sequence of peaks within these three areas, with a high peak in the nonpolar (hydrophobicity/lipophilicity) areas, showing the affinity of nonpolar molecules. The *σ* regions beyond +0.01 e.nm^−2^ and behind −0.01 e.nm^−2^ are strongly polar with the potential to form hydrogen bonds (HB). The region surrounded within the ±0.01 e.nm^−2^ is considered nonpolar. The high polarized charge at –0.002 e.nm^−2^ corresponds to the hydrocarbon chain and the methyl fragment. These two slight peaks in the polar regions underline the ALA molecules’ capacity through their oxygen or hydrogen atoms to form hydrogen bonds with ILs. This implies that the ALA molecules can behave as donors and acceptors of hydrogen bonds. 

Figure 4 determines the sigma potential of the ALA compounds. By estimating the total energy or chemical potential, the sigma potential is calculated. The total energy is formed up of two parts: (a) restoring free energy, which aims to restore the molecule to its original and hence, the essential energy would be positive in nature, and (b) abandoned hydrogen bonding energy (negative in nature) due to the hydrogen bond interaction between the hydrogen bond donor sectors and the hydrogen bond acceptor sectors. A net adverse charge indicates the dominance of hydrogen bonding, whereas a net positive charge indicates the dominance of free energy restoration [38]. Consequently, on the following cut-off values, the COSMO-RS histogram is qualitatively split into three major regions: hydrogen bond donor (*σ* < −0.01 e.nm^−2^), hydrogen bond acceptor (*σ* > + 0.01 e.nm^−2^), and nonpolar region (−0.01 < *σ* < 0.01 e.nm^−2^). The negative values of the sigma potential (Y-axis) are a sign of the interaction between the ALA molecules and the ILs.

### 2.1. Capacity Values of ILs toward the ALA Molecules

**Figure 5:**
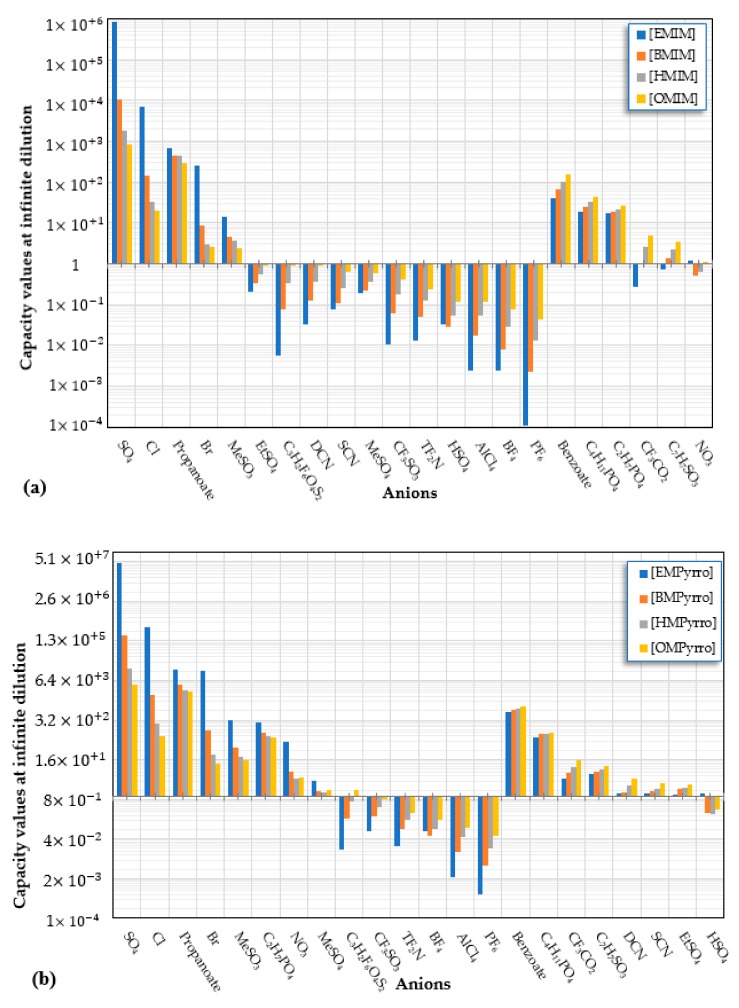

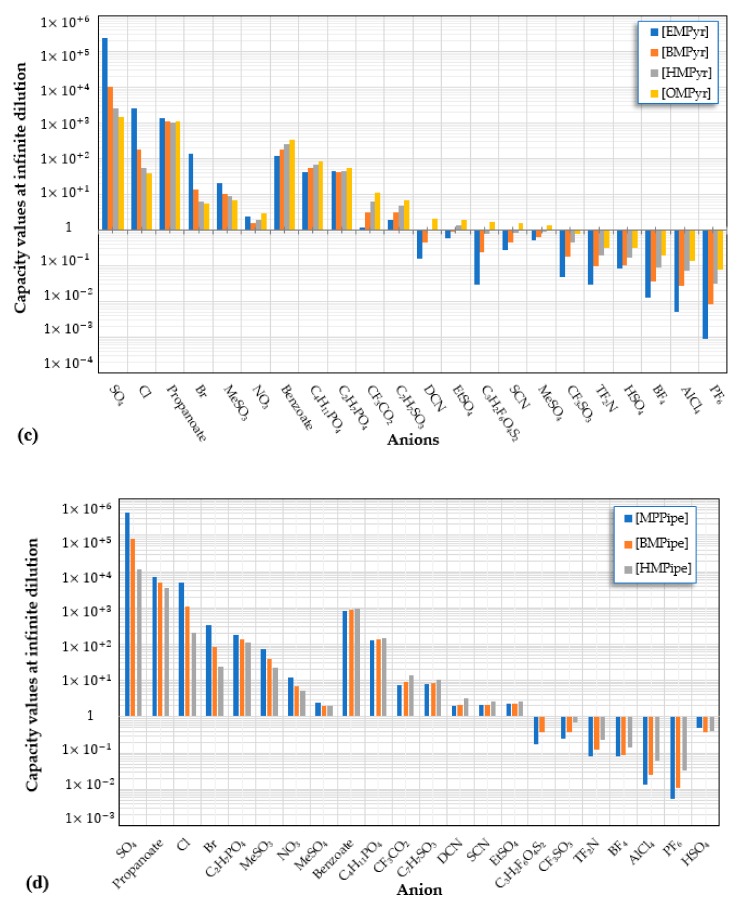

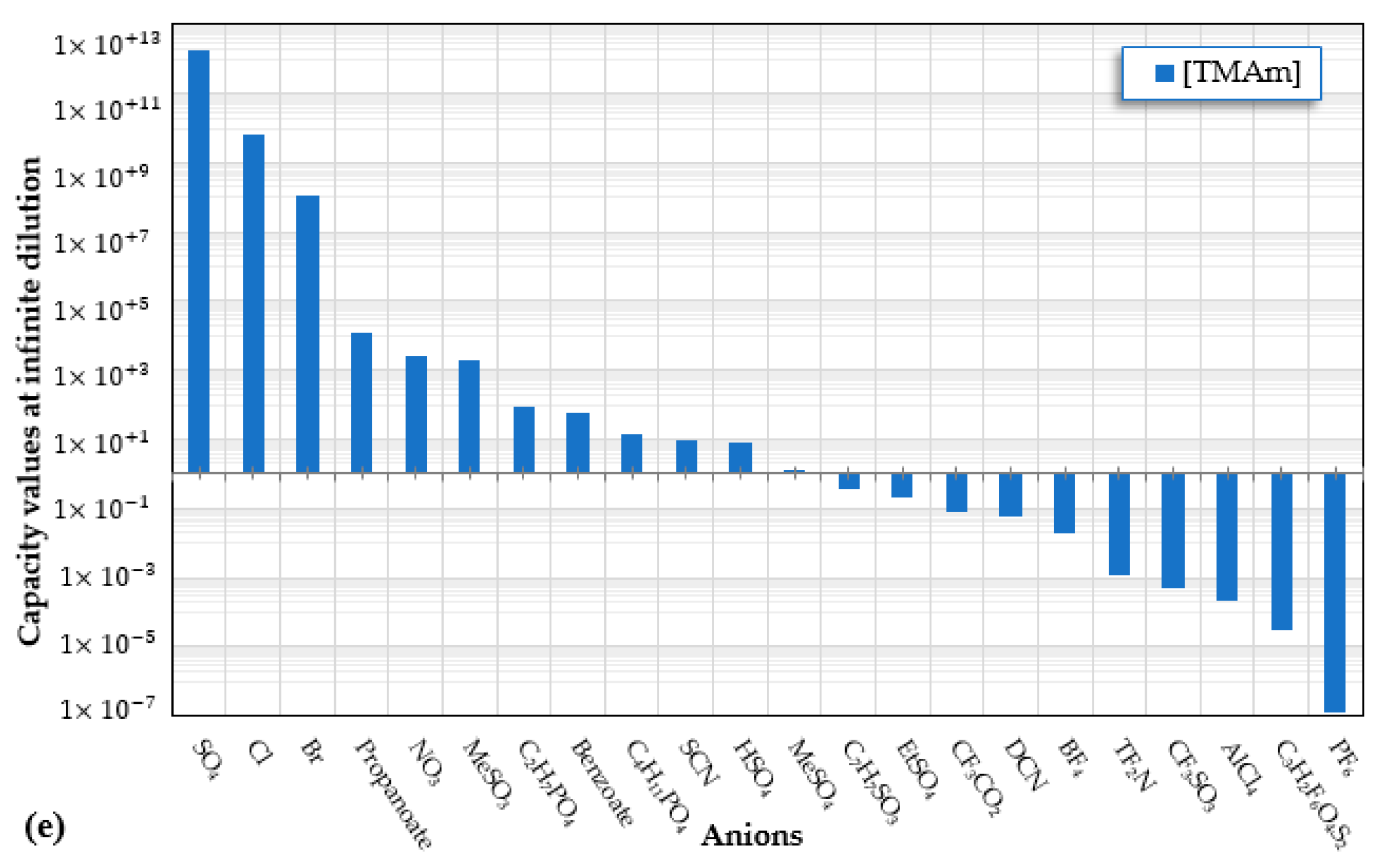
COSMO-RS predicted infinite dilution capacity values (Y axis) of ionic liquids (ILs) comprising (**a**) Imidazolium; (**b**) Pyridinium; (**c**) Pyrrolidinium; (**d**) Piperidinium; (**e**) Teramethyl ammonium based cations alkyl chain length with 22 anions (X axis) at 25 °C for ALA extraction.

Capacity values were screened at 25 °C for the five kinds of cation-based ILs, comprising imidazolium, pyridinium, pyrrolidinium, piperidinium, and tetramethyl ammonium with 22 anion varieties (Figure 5a–e). Figure 5 indicates the behavior of cations when combined with various anions. The data demonstrates the capacity values of ALA extraction. For instance, in the case of imidazolium-IL, the suggested anions comprise SO_4_^−2^, Cl^−^, propanoate and Br^−^. The outcomes are accompanied by the recommendations for the selection of appropriate ILs to be used in the extraction of ALA compounds as a part of solid-liquid extraction. On the other hand, anions such as methyl sulfate, methyl thiosulfate, DCN^−^, SCN^−^, HSO_4_^−^, AlCl_4_^−^, NO_3_^−^, BF_4_^−^, and PF_6_^−^ were not on the list of preferable options. Those anions have less ability to form hydrogen bonds with the algae biomass except for sulfate derived anions.

A similar trend was also observed with EMPyrro. However, it can be seen that the cations in group (b) were better suited with NO_3_^−^, DCN^−^, MeSO_3_^−^, EtSO_4_^−^, and benzoate, in addition to the SO_4_^−2^, Cl^−^, propanoate, and Br. Likewise, MPPipe and cations in group (d) as well as TMAm displayed a similar trend in the capacity. It was also observed that pyrrolidinium and pyridinium as well as tetramethyl ammonium cations behaved similarly. Tetramethyl ammonium has poor nitrogen atoms that could easily accept electrons from higher electronegativity atoms to form bonds with small anions such as SO_4_^−2^, Cl^−^, propanoate, and Br^−^. This also holds true in the case of pyrrolidinium and pyridinium cations. On the other hand, piperidinium cation with nitrogen surrounded by more alkyl groups could properly favor the higher electronegative anions.

#### 2.1.1. Effect of Carbon Chain Length of Cation-Based ILs on the ALA Extraction Capacity

Different cations with different lengths of alkyl chain were examined in this study to comprehend their efficacy in the ALA extraction. The studies cations include imidazolium, pyridinium, pyrrolidinium, piperidinium, and tetramethyl ammonium. The rise in the alkyl chain length of the imidazolium cations is perceived to be associated with a gradual enhancement in the extraction ability and capacity values of the ILs. A few exceptions were detected when the cation was combined with SO_4_^−2^, Cl^−^, Br^−^, NO_3_^−^, methylsulfonate, and propanoate, where the highest capacity was recorded with [EMIM]_2_SO_4_. The capacity of the imidazolium based ILs is found to be in the order of [EMIM] > [BMIM] > [HMIM] > [OMIM]. The exact same trend is also observed when the capacity is compared with pyridinium ILs, with the largest capacity associated with [EMPyr]_2_SO_4_.

On the other side, an enhance in the alkyl chain length attached to the cation 1-alkyl-1-methyl pyrrolidinium has less influence on the extraction capacity. Increasing the duration of the alkyl chain, however, does not enhance the ability considerably. With most of the anions used in this job, this proclamation is accurate. However, exceptions are observed with SO_4_^−2^, Cl^−^, Br^−^, NO_3_^−^, propanoate, and dimethylphosphate where the capacity drops for the longer alkyl chains. Nevertheless, larger capacity is denoted in the latter. The highest ability is found again with the brief alkyl chains ([EMPyr]) and inorganic anions instead of the organic counterparts. This could be easily explicated by the reality that the organic molecules are bigger in size and that Van der Waals displays forces between the molecules that obstruct the exchange of anions. 

The trend for cation piperidinium is quite variable as it fluctuates according to the anion. With 1-methyl-1-propyl-piperidinium ([PMPipe]), the extraction capability rises compared to butyl and hexyl, indicating that the longer the alkyl chain, the more reduced the IL capacity values. This seems to be the situation of the anions used except for benzoate where the hexyl chain ([HMPipe]) has the highest ability. Based on the results, [PMPipe] has the highest extraction capacity, especially with SO_4_^−2^, Cl^−^, and propanoate. Likewise, tetramethyl ammonium displays a similar capacity to piperidinium based ILs with SO_4_^−2^, Cl^−^, Br^−^, and propanoate. 

A main advantage of using ILs is the adjustability of their physicochemical characteristics by altering the cation or anion species in the extraction and separation scheme. This allows to tailor ILs to target particular separation compounds. It goes beyond also, as imidazolium and pyridinium cation based IL are frequently applied in metal ions, organic compounds, and biomolecule extraction forming various kinds of matrices [39]. Moreover, inorganic anions have been noted to have greater capacity values than the alkyl anions. The interaction between the cation and the anion is supposed to result from the lack of any alkyl chain in the anion. Therefore, this makes the interaction (i.e., cation-anion) stronger than the ALA molecules interaction. Hence, their capacity values for the extraction is dropping. In addition, as the length of the alkyl chain rises, the charge density on the cation decreases and the molar volume rises. This is because the ions are unable to pack efficiently due to the flexible alkyl chain; this leads to a reduction in density. It is likely that a mixture of development in the alkyl chain length (and the related flexibility) may limit the motion of one element of the IL past another together with the rise in dispersion forces resulting from a rise in molecular volume [40]. 

It was recognized that ILs with long alkyl chains cations can considerably enhance the solubility of oleanolic acid in aqueous media and thus compete with the solubility provided by molecular organic solvents such as chloroform [41]. Furthermore, the impact of the IL cation, which is perhaps dominated by interactions between the π―π, so, should be considered in extractions, however, it cannot be blindly generalized either [42]. In addition, the ALA hydroxyl groups can readily replace small anions. However, this only seems true for the brief alkyl chains where the impact seems to disappear by raising the length of the chain. It also appears that the highest capability is observed with EMIM instead of other ILs based on imidazolium and [EMPyr] and [EMPyrro] instead of the longer chain cations.

The 1-alkyl-3-methylimidazolium cations, [C*_n_*C_1_IM]^+^ are indeed the most investigated, while [BF_4_]^−^, [PF_6_]^−^ (both water-unstable), Cl^–^, [Tf_2_N]^−^ (fragile biodegradable and toxic), and [C*_n_*CO_2_]^−^ are the most broadly studied anions. In a study by [43], the extraction of fatty acids from cyanobacteria biomass was conducted. Only [BMIM]Cl was able to dissolve this marine biomass from the two ILs studied, particularly at greater temperatures. Some other works from the same research group reported compound co-extraction by adopting a similar technique, e.g., the co-recovery of bio-oil and phorbol ester from jatropha biomass using [EMIM][CH_3_SO_4_] and methanol as cosolvents [44]. Lipid extraction efficiency from *Chlorella vulgaris* microalgae biomass has been successfully enhanced through the utilization of [BMIM][CF_3_SO_3_]-methanol (12.5%–19.0% of lipids extracted) [24]. Their findings were similar with those observed through the application of a standard technique used by Bligh and Dyer (10.6%–11.1% of the extracted lipids). The same research group similarly described the application of ultrasound-assisted irradiation techniques for the lipid extraction. The usage of [BMIM][CH_3_SO_4_] showed higher extraction amounts of lipids from *Chlorella vulgaris* microalgae biomass (75 mg g^−1^ of dry cell weight and 47 mg g^−1^ of dry cell weight, with and without ultrasonic irradiation) compared with the two other standard techniques, Bligh and Dyer’s method (29 mg g^−1^ of dry cell weight) and Soxhlet (21 mg g^−1^ of dry cell weight) [23]. In addition, also obtained was carotenoid, a fat-soluble pigment using [EMIM][C_2_H_5_SO_4_]. This IL has demonstrated the highest output for astaxanthin among the investigated ILs [45]. It was demonstrated that π···π stacking governs the selective adsorption of PUFAs and ethyl esters from fish oil. Those with aromatic rings disclosed favored selectivity for the polyunsaturated compounds with 11 ILs studied [46].

#### 2.1.2. Effect of Anion-Based ILs on the ALA Extraction Capacity

In this research, 22 anions were explored in combination with different cations. Sulfate and chloride showed the greatest ability for the extraction of ALA compound. Though the capacity values for the tetramethyl ammonium and SO_4_^2−^ pair followed by bromide are the greatest, a general decision cannot be drawn based on the simulation results only, as, in this case, there was only one ammonium-based cation tested. All an all, poor capacities are recognized when the cations [C_n_MPyrro], [C_n_MPipe] and [C_n_MPyr] are involved regardless of the anion type—particularly with an increase in the alkyl chain length. However, for [C_n_MIM], the largest capacities are seen with SO_4_^2−^ followed by chloride, with the short-chain such as in [EMIM]. Moreover, poor capacity values were recorded with other anions as well. With [C_n_MPyr], the same trend is seen with [EMPyr] in the order of SO_4_^−2^ > Cl^−^ > propanoate. Likewise, [EMPyrro] has a similar trend when paired with SO_4_^−2^, Cl^−^, Br^−^, and propanoate, respectively, where the values drop in the longer alkyl chain. Similarly, [PMPipe] presents a similar trend in the order of SO_4_^−2^ > Cl^−^ > propanoate, although the cyclic nature of the cation might slightly decrease the capacity. 

**Figure 6:**
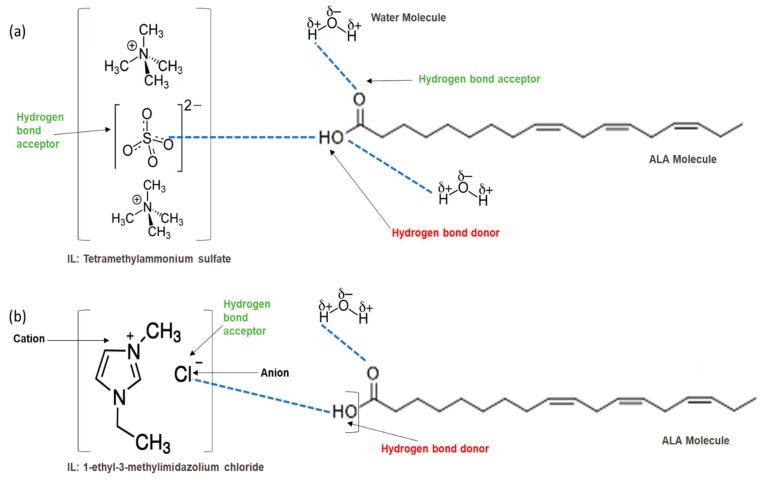
Suggested bonding during the extraction of ALA molecules using ILs; (**a**) IL: Tetramethyl ammonium sulfate [TMAm]_2_[SO_4_], (**b**) IL: 1-ethyl-3-methylimidazolium chloride [EMIM]Cl.

Based on the outcomes, the hydrogen bond (S = O ... H) between the cation and the anion pair could be speculated to be powerful. This is in line with the overall assumption that S = O is a very excellent proton acceptor when forming H-bonds with the ALA’s OH group, which eventually facilitates the extraction process. This portent may be performed in the case of imidazolium-based IL with SO_4_^−2^ as the anion, and with other cations with lone pairs of electrons. The different changing patterns in the strength of hydrogen bond propose the probable existence of selective interactions among anion, water, and acidic protons on the imidazolium ring [47]. The illustration of Figure 6b shows [EMIM]Cl interactions during ALA extraction. As the extraction is performed in aqueous mixture of IL and water, it is expected that hydrogen bonding might occur between the hydroxy group of ALA and chloride from [EMIM]Cl which facilitate the extraction due to the high electronegativity of the chloride. There is a possibility of hydrogen bonding between the water molecules in the mixture and the carbonyl group of ALA molecules, which might enhance the extraction due to the high polarity of acid and water. An example of the extraction procedure is demonstrated in Figure 6.

Organic anions demonstrate bad capacities for the extraction of ALA. This is true of all the researched organic anions. With all cations with short alkyl chains, the exception is propanoate. On the other hand, inorganic anions, (i.e., SO_4_^−2^ and Cl^−^) could be suitable candidates if combined with cations with short alkyl chains such as [EMIM], [EMPyrro], [EMPyr], and [PMPipe]. Tetramethyl ammonium-IL display the highest capacity value when the cation is paired with SO_4_^−2^, Cl^−^, and Br^−^. Furthermore, tetramethyl ammonium cation has a positively charged nitrogen as all electrons are occupied with methyl groups. Small anions such as sulphate, chloride, and bromide, therefore, thrive to achieve their outer energy level with eight electrons and thus tend to lose the electron from the anion’s nucleus to reach a stable state (Figure 6). 

**Figure 7:**
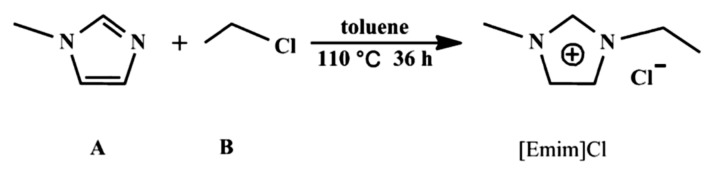
A schematic diagram for the synthesis of 1-ethyl-3-methyl imidazolium chloride ([EMIM]Cl) from N-methyl imidazole (**A**) and chloroethane (**B**). Adapted with permission from Mu et al. [48] The Journal of Physical Chemistry A. Copyright (2017) American Chemical Society.

In fact, the reaction does not happen easily. In reality, the system described in Figure 7 is almost followed. However, the goal is to show the electrons available in the ILs.

It could be anticipated that the anion may be in a more stable situation in the event of larger alkyl groups, which may hinder its extraction ability. This is because longer alkyl groups are strongly connected to Van-der Walls forces, a force that grows even stronger in the length of the carbon chain. In this context, Cheong et al. suggested the correlation of n-3 PUFA extraction and structure of ILs (aromatic/delocalized cation) for the effective extraction of PUFA [46]. The short alkyl chain was used in a recent study [49]. Two types of ILs were used in the extraction of PUFA-containing lipids from *Thraustochytrium sp.* (T18) i.e., imidazolium (1-ethyl-3-methylimidazolium ethyl sulfate [EMIM][EtSO_4_]) IL and phosphonium (tetrabutyl phosphonium propanoate [P_4444_][Prop]). This is in line with the outcomes of COSMO-RS, which demonstrate that propanoate is indeed an outstanding extraction alternative when coupled with cations having short alkyl chain lengths like ethyl.

In a study [24], the lipid was extracted from dry *Neochloris oleoabundans* microalgae utilizing the various ILs. Then the lipid extraction yield was obtained using [BMIM] as cation-based IL combined with anions: MeSO_4_^−^, BF_4_^−^, DCN^−^, and Cl^−^, respectively. The results revealed that the anionic structure of the ILs are directly linked to the extraction efficiencies of lipids. Previous literature showed that, by and large, the lipid extraction yield enhanced with diminishing polarizability/dipolarity and with accelerating acidity values of ILs; nevertheless, water immiscible and hydrophobic ILs established a low extraction efficiency, while water-miscible and hydrophilic ILs are associated with high extraction performance [50]. [BMIM]Cl has also been determined to have a potential to damage the membrane structure of *Skeletonema marinoi* and *Phaeodactylum tricornutum* microalgae. Thus, distinct microalgae cell wall structures (i.e., cellulose, glycoprotein, silica, and peptidoglycan) may play a critical role in IL species selection. The *Neochloris oleoabundans* cell membrane structure rich in fibrous constructions which may be more prone to [BMIM][MeSO_4_] [46,47,48]. It was suggested that it disrupts the fiber bundle structure in the cell wall, which in turn promotes the release of lipids from the microalgae cell membranes [51]. Bonding between the OH groups of the molecules (to be extracted) and the anion of the ILs has also been recorded. It was also suggested that the reaction of the polar covalent molecules (such as methanol and water) could significantly disturb the cytomembrane and thus enhance the effectiveness of extracting the lipid from the biomass [52]. It can be deduced that enhancing the alkyl chain length of cations would result in lower selectivity and poor extraction capability of ILs. Though, similar behavior cannot be justified for the cations.

**Figure 8:**
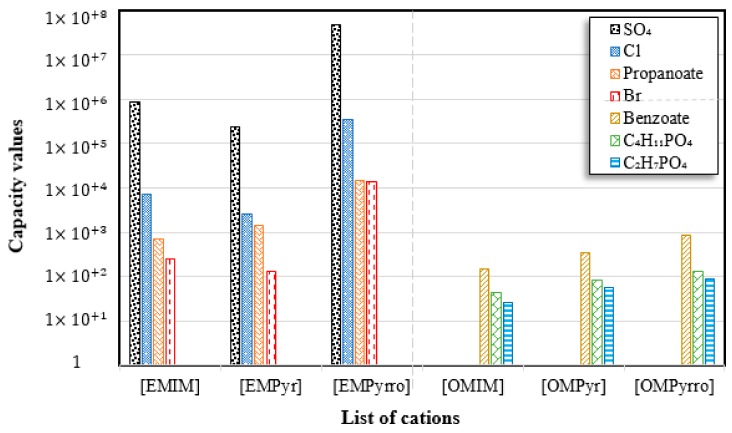
Comparison of imidazolium, pyridinium, and pyrrolodinium cations with various anions in terms of capacity values for ALA extraction at 25 °C. [EMIM]: 1-ethyl-3-methyl imidazolium, [EMPyr]: 1-ethyl-3-methyl pyridinium, [EMPyrro]: 1-ethyl-1-methyl pyrrolidinium, [OMIM]: 1-octyl-3-methyl imidazolium, [OMPyr]: 1-octhyl-3-methyl pyridinium, [OMPyrro]: 1-methyl-1-octyl pyrrolidinium.

Figure 8 compares the capacity values of different cations with various anions. It is seen that the largest values have been recorded in the order of [EMPyrro]SO_4_ > [EMIM]SO_4_ > [EMPyrro]Cl > [EMPyr]SO_4_ > [EMPyrro]propanoate > [EMPyrro]Br > [EMIM]Cl. On the other hand, [OMIM], [OMPyr], and [OMPyrro] have shown lower capacity values relative to shorter alkyl chains. However, the mentioned cations displayed high capacity with chloride as an anion. This might seem logical as Cl^−^ is a relatively small anion compared to other anions tested in the study. As initially discussed, it is seen that the trend supports the hypothesis that cations with short alkyl chains are better options for the extraction processes.

The IL-assisted extraction of vital oil from *Polygonum minus* was researched and contrasted on the output of extracted vital oil for different handling techniques (e.g., microwave, ultrasonic, reflux, and mechanical stirring). ILs with various anions (i.e., Tf_2_N^−^, Cl^−^, and acetate), and cations (i.e., [AMIM], [BMIM], and [HMIM]) were examined. Among the studied ILs, 1-ethyl-3-methylimidazolium acetate ([EMIM]Ace) was seen to be most effective using a microwave-assisted extraction process [53].

### 2.2. Experimental Validation of COSMO-RS Prediction for the ALA Extraction

**Figure 9:**
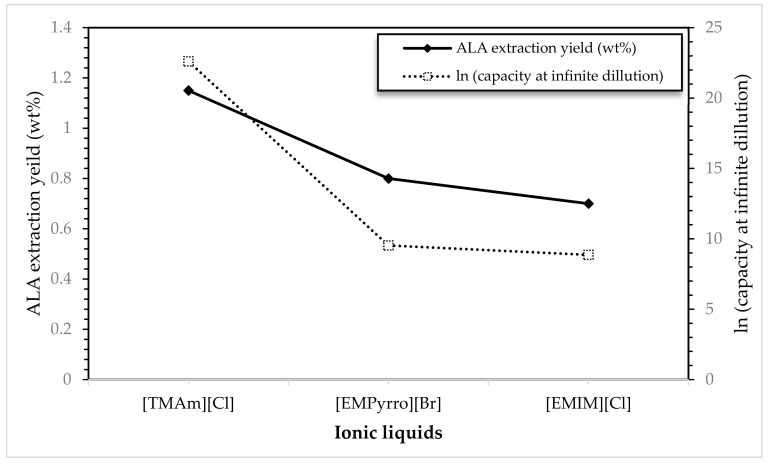
Comparison of the capacity values of selected ILs toward ALA compound predicted by COSMO-RS and the experimental ALA extraction yield (%) from *Nannochloropsis sp.* microalgae. [TMAm][Cl]: tetramethyl ammonium chloride, [EMPyrro][Br]: 1-ethyl-1-methyl pyrrolidinium bromide, [EMIM][Cl]: 1-ethyl-3-methyl imidazolium chloride.

Three types of ILs were selected for the validation study and an experimental evaluation was performed to examine whether the ILs with higher extraction capacities as predicted by COSMO-RS are also able to practically increase the extraction of ALA from microalgae. The selected ILs for validation are namely [EMIM][Cl], [TMAm][Cl], and [EMPyrro][Br] and the selection of them are due to their high capacity values and their commercial availability. According to the results shown in Figure 9, it is clear that as the extraction yield of ALA is positively correlated with the capacity of the corresponding ILs. The logarithmic values of capacities were found about 22.60, 9.52, and 8.86 in order of higher to lower for [TMAm][Cl], [EMPyrro][Br], and [EMIM][Cl], respectively. The extraction yields were, in fact, found with a comparable tendency of higher to lower corresponding to the similar order of ILs i.e., 1.15, 0.8, and 0.7 wt% related to [TMAm][Cl], [EMPyrro][Br], and [EMIM][Cl], respectively. It can be eventually concluded that the lower/higher level of ALA extraction can be defined according to the lower/higher values of COSMO-RS predicted capacities of the ILs hence the model seemed to be accurate and practically implementable.

## 3. Methodology

### 3.1. COSMO-RS Computational Details and Calculations

COSMOtherm C2.1 is a software package based on the COSMO-RS principle for calculating and/or estimating thermodynamic properties. The Conductor-like Screening Model for Real Solvent (COSMO-RS) created by [54] is regarded as a strong molecular description and solvent screening technique based on a quantum-chemical strategy. COSMO-RS combines quantum chemical factors (COSMO) and statistical thermodynamics (RS) to predict the thermodynamic properties with low experimental information. Using COSMO-RS, we used a six-step procedural path to estimate IL capability values. Initially, the Turbo mole Version 7.1 software draws all structures of cations, anions, and IL pairs (ion pair). The geometry optimization is then performed using the level of identity resolution (DFT-RI) functional density theory. The Triple Zeta Valence Potential (TZVP) is used in conjunction with the PB-3LYP base set to work out the complicated system of cation and anions [55,56]. Next, COSMO-RS calculates the ALA molecules’ sigma surfaces. The software then calculates the sigma profile of each species in the third stage (the equations are available elsewhere) [24]. Based on the *σ*-profiles, the *σ*-potential of a molecule is estimated in the next step. The temperature and pressure were constant at 25 °C (environmental temperature) and 1 atm, respectively, in this research. In the fifth step, COSMO-RS calculates the coefficient of activity associated with the sigma potential at infinite dilution (Equation (1)). Finally, COSMO-RS calculated the capacity values of the ILs to extract the ALA molecules using Equation (2) below.

(1) $$Ac_{s}^{x_{i}}=exp\left\{\frac{\mu_{s}^{x_{i}}-\mu_{x_{i}}^{x_{i}}}{RT}\right\},\;$$

(2) $$\mathrm{Capacity}\mathrm{values}={\left[\frac{1}{Ac}\right]}^{IL\;phase}.$$

In Equation (1), $\mu_{s}^{x_{i}}$ is the potential of the solvent *s*, and $\mu_{x_{i}}^{x_{i}}$ is the potential of the pure component $x_{i}$.

The lists of cations and anions for the ILs studied in this work are represented in Table 1 and Table 2, respectively.

### 3.2. Material and Methods of Experimental Validation

The frozen dried biomass (*Nannochloropsis* sp.) was bought and transported from Longevity Superfoods (Utah, USA batch no: 32490). The powdered biomass was kept wrapped in the package and stored while not being used in experiments. Hexane, chloroform (>99.8%), and methanol were supplied from R&M Chemicals (Kuala Lumpur, Malaysia) in analytical grade. The ionic liquids including 1-ethyl-3 methyl imidazolium chloride ([EMIM][Cl], 94.5%), tetramethyl ammonium chloride ([TMAm][Cl], ≥99%) and 1-ethyl-1-methyl pyrrolidinium bromide ([EMPyrro][Br], 99%) were purchased from Merck (Kenilworth, New Jersey, USA) and utilized without any additional purification. The microwave oven reactor (Samsung, ME711K) was purchased in Malaysia under the property of Universiti Putra Malaysia, laboratory of combustion, faculty of engineering.

### 3.3. Microwave Assisted Extraction (MAE) of Lipid with ILs

The total lipid was extracted using the domestic microwave assisted extraction with 700 W of energy at a frequency of 2.45 GHz. The method was adapted from previous work by [57]. The dry microalgae biomass (0.5 g) was appropriately mixed with distilled water (3.3 %wt) and ILs (2 g). Samples were extracted at 90 °C for 25 min. After microwave heating, methanol and chloroform were added to the sample and underwent the phase separation using a centrifuge (4000 rpm, 10 min). Then, lipids were recovered from chloroform phase and finally separated through evaporation. 

The fatty acid methyl esters (FAMEs) were produced from the extracted *Nannochloropsis* sp. lipids through the transesterification method adapted from previous literature [57,58]. The produced FAMEs which contained ALA composition were then separated and sent for gas chromatography (Agilent 6890 GC, USA) equipped with a flame ionization detector (FID) analysis.

## 4. Conclusions 

In this research, various ILs were explored for their capacity values in ALA extraction through simulation using COSMO-RS. However, other important variables such as toxicity, thermal stability, volume of anions, energy interaction between cation and anion (in addition to recyclability and solubility of ILs) should also be taken into account in order to identify the most practically efficient ILs. Short alkyl chain cations and inorganic anions are preferable in the extraction process based on our simulation outcomes. In comparison to the other studied ILs, sulphate-, chloride-, and bromide-based ILs were found to have greater extraction capacities, although it was revealed that propanoate has significant capacity when coupled with ethyl group cations. This study also tested three selected ILs with the highest predicted extraction capacities from COSMO-RS in extracting ALA from microalgal biomass. It was found that the extraction yields gave a similar trend with the COSMO-RS predicted capacity values. This research offers sound understanding to enable scientists to define appropriate ILs with potential for ALA extraction from microalgae biomass. By using high capacity ILs as solvents and/or co-solvents, it would not only be possible to achieve a high amount of extraction efficiency but could also contribute to a more sustainable method with integrated minimization and/or avoidance of the use of dangerous organic solvents overall. 

## Figures and Tables

**Table 1:** The list of cations tested in this research.

No.	Name of Anions	Acronym	Chemical Structures
1	1-ethyl-3-methyl imidazolium	[EMIM]	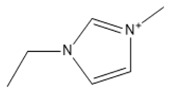
2	1-butyl-3-methyl imidazolium	[BMIM]	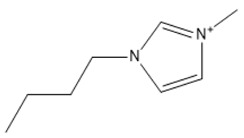
3	1-hexyl-3-methyl imidazolium	[HMIM]	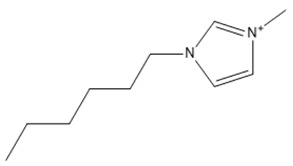
4	1-octyl-3-methyl imidazolium	[OMIM]	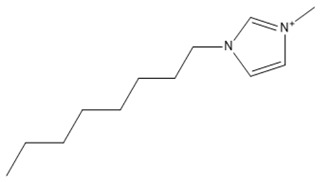
5	1-ethyl-3-methyl pyridinium	[EMPyr]	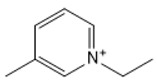
6	1-butyl-3-methyl pyridinium	[BMPyr]	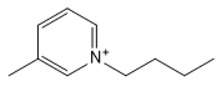
7	1-hexyl-3-methyl pyridinium	[HMPyr]	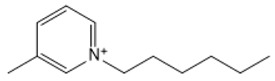
8	1-octhyl-3-methyl pyridinium	[OMPyr]	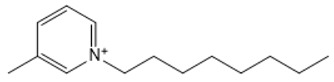
9	1-ethyl-1-methyl pyrrolidinium	[EMPyrro]	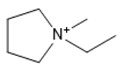
10	1-butyl-1-methyl pyrrolidinium	[BMPyrro]	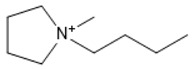
11	1-hexyl-1-methyl pyrrolidinium	[HMPyrro]	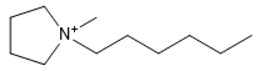
12	1-methyl-1-octyl pyrrolidinium	[MOPyrro]	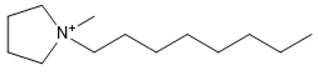
13	1-methyl-1-propyl piperidinium	[MPPipe]	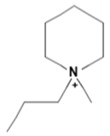
14	1-butyl-1-methyl piperidinium	[BMPipe]	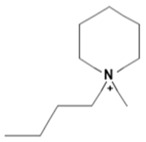
15	1-hexyl-1-methyl piperidinium	[HMPipe]	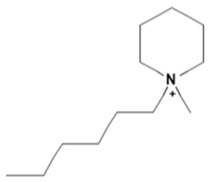
16	Tetramethyl ammonium	[TMAm]	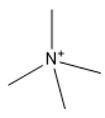

**Table 2:** The list of anions tested in this research.

No.	Name of Anions	Acronym/Chemical Structure Formula	Chemical Structures
1	Chloride	Cl^−^	-
2	Bromide	Br^−^	-
3	Tetrafluoroborate	[BF_4_]^−^	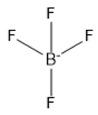
4	Hexafluorophosphate	[PF_6_]^−^	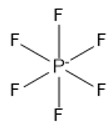
5	Nitrate	[NO_3_]^−^	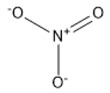
6	Dicyanamide	[DCN]/[C_2_N_3_]^−^	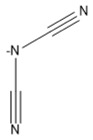
7	Tetrachloro aluminate	[AlCl_4_]^−^	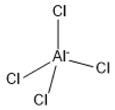
8	Thiocyanate	[SCN]^−^	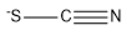
9	Dimethylphosphate	[C_2_H_6_PO_4_]^−^	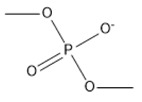
10	Diethylphosphate	[C_4_H_10_PO_4_]^−^	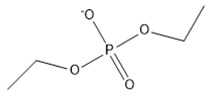
11	Benzoate	[C_7_H_5_O_2_]^−^	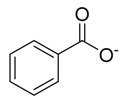
12	Methanesulfonate	[CH_3_SO_3_]^−^	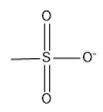
13	Toluene-4-sulfonate	[C_7_H_7_SO_3_]^−^	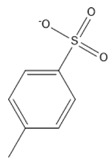
14	Trifluoro methane sulfonate	[CF_3_SO_3_]^−^	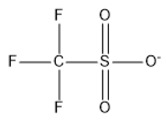
15	Sulfate	[SO_4_]^2−^	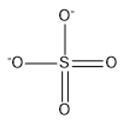
16	Hydrogen sulfate	[HSO_4_]^−^	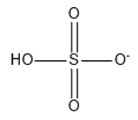
17	Ethyl sulfate	[C_2_H_5_SO_4_]^−^	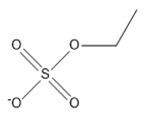
18	Methyl sulfate	[CH_3_SO_4_]^−^	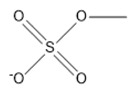
19	Propanoate	[C_3_H_5_O_2_]^−^	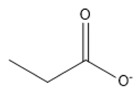
20	Bis(trifluoromethylsulfonyl)methane	[C_3_H_2_F_6_O_4_S_2_]^−^	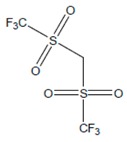
21	Trifluoro acetate	[CF_3_CO_2_]^−^	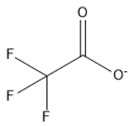
22	Bis(trifluoromethylsulfonyl)amide	[Tf_2_N]]/[C_2_F_6_NO_4_S_2_]^−^